# Immunomodulation Evidence of Nanostructured Recombinant Proteins in Salmonid Cells

**DOI:** 10.3390/ani14060844

**Published:** 2024-03-09

**Authors:** Débora Torrealba, Daniela López, Patricio Zelada, Nicolás Salinas-Parra, Paula Valenzuela-Avilés, Elena Garcia-Fruitós, Anna Arís, Luis Mercado, Claudia Altamirano, José Gallardo-Matus

**Affiliations:** 1 Instituto de Ciencias de la Ingeniería, Universidad de O’Higgins, Avenida Libertador Bernardo O’Higgins 611, Rancagua 2841959, Chile; 2 Laboratorio de Genética y Genómica Aplicada, Escuela de Ciencias del Mar, Pontificia Universidad Católica de Valparaíso, Avenida Universidad 330, Valparaíso 2373223, Chilenicolas.salinas@pucv.cl (N.S.-P.); jose.gallardo@pucv.cl (J.G.-M.); 3 Programa de Doctorado en Biotecnología, Pontificia Universidad Católica de Valparaíso, Universidad Técnica Federico Santa María, Valparaíso 2373223, Chile; 4 Escuela de Ingeniería Bioquímica, Pontificia Universidad Católica de Valparaíso, Avenida Brasil 2085, Valparaíso 2373223, Chile; patricio.zelada.c@gmail.com (P.Z.); claudia.altamirano@pucv.cl (C.A.); 5 Departamento de Biología Molecular y Biotecnología, Instituto de Investigaciones Biomédicas, Universidad Nacional Autónoma de México, AP 70228, Ciudad de México 04510, Mexico; 6 Programa de Doctorado en Ciencias Bioquímicas, Universidad Autónoma de México (UNAM), Ciudad de Mexico 04510, Mexico; 7 Grupo de Marcadores Inmunológicos en Organismos Acuáticos, Instituto de Biología, Pontificia Universidad Católica de Valparaíso, Avenida Universidad 330, Valparaíso 2373223, Chile; luis.mercado@pucv.cl; 8 Department of Ruminant Production, Institute of Agriculture and Food Research (IRTA), 08140 Caldes de Montbui, Spain; elena.garcia@irta.cat (E.G.-F.); anna.aris@irta.cat (A.A.); 9 Center of Interventional Medicine for Precision and Advanced Cellular Therapy (IMPACT), Santiago 7550000, Chile

**Keywords:** inclusion bodies, antimicrobial peptides, cytokines, aquaculture, rainbow trout, Atlantic salmon, immunostimulants

## Abstract

**Simple Summary:**

In salmonid aquaculture, new prophylactic methods are needed to combat infectious diseases. Bacterial inclusion bodies are nanostructured particles of recombinant proteins formed during recombinant protein production processes. They are mainly composed of functional recombinant proteins, highly stable without the need for encapsulation, and can be produced through a cost-effective bacterial process. Different applications of these nanoparticles have been described, including their ability to enhance immune function. These characteristics make them very attractive for use in aquaculture. This study explored the production of three immune-related proteins as inclusion bodies and evaluated their ability to stimulate the immune response in the rainbow trout RTS11 cell line. In addition, we characterized the scaling-up of inclusion bodies production by comparing two different scale systems. We successfully produced three proteins as inclusion bodies that are able to activate the immune response in a cell line. Regarding the scaling-up of production, we observed that the inclusion bodies produced in a bioreactor were the smallest and had a greater ability to activate the immune response in the RTS11 cell line than in the same protein produced in a shaken flask. More studies are needed to characterize the immune response activated by these inclusion bodies and the optimal production conditions in bioreactors for generating inclusion bodies, such as media, fed-batch production, and mechanical bacterial lysis.

**Abstract:**

Recent studies have demonstrated that immune-related recombinant proteins can enhance immune function, increasing host survival against infectious diseases in salmonids. This research evaluated inclusion bodies (IBs) of antimicrobial peptides (CAMP^IB^ and HAMP^IB^) and a cytokine (IL1β^IB^ and TNFα^IB^) as potential immunostimulants in farmed salmonids. For this purpose, we produced five IBs (including iRFP^IB^ as a control), and we evaluated their ability to modulate immune marker gene expression of three IBs in the RTS11 cell line by RT–qPCR. Additionally, we characterized the scale-up of IBs production by comparing two different scale systems. The results showed that CAMP^IB^ can increase the upregulation of *tnfα*, *il1β*, *il8*, and *il10*, HAMP^IB^ significantly increases the upregulation of *tnfα*, *inos*, and *il10*, and IL1β^IB^ significantly upregulated the expression of *tnfα*, *il1β*, and *cox2*. A comparison of IL1β^IB^ production showed that the yield was greater in shake flasks than in bioreactors (39 ± 1.15 mg/L and 14.5 ± 4.08 mg/L), and larger nanoparticles were produced in shake flasks (540 ± 129 nm and 427 ± 134 nm, *p* < 0.0001, respectively). However, compared with its shake flask counterpart, the IL1β^IB^ produced in a bioreactor has an increased immunomodulatory ability. Further studies are needed to understand the immune response pathways activated by IBs and the optimal production conditions in bioreactors, such as a defined medium, fed-batch production, and mechanical bacterial lysis, to increase yield.

## 1. Introduction

Global aquaculture production has grown steadily since 1950, registering 638,457 tons instead of 122.6 million tons in 2020 [1]. In particular, the export of Atlantic salmon and rainbow trout accounted for 18.4% of the value of all aquatic products exported in 2020, up from 5.1% in 1976 [2]. One of the main challenges in maintaining the sustainability of this industry is the control of infectious diseases, which are aggravated by overcrowding culture systems. Disease control and its negative impact on production are estimated to cause economic losses of more than USD 10 billion on a global scale per year [3]. Consequently, new and diverse alternatives to treat and control diseases, including vaccines (e.g., protein-based vaccines and autogenous vaccines), immunomodulators (e.g., chimeric proteins, phytogenic compounds, and hormones), and breeding programs, have been explored in recent years to meet this growing need in aquaculture [4,5,6,7,8,9,10,11]. Immunomodulators play a role in enhancing immune function and improving host performance against pathogens [12,13]. There are diverse categories of immunomodulators for fish, such as probiotics, phytogens, chemicals, hormones, metals, nutritional factors, yeast hydrolysate, and immune molecules [4]. They are promising alternatives for preventing and controlling infectious diseases because they can enhance the immune system, protect the host against a wide range of pathogens, can be added to food, and are alternatives to vaccines in fish [4,14].

Proteins from the host immune system, such as antimicrobial peptides, cytokines, chemokines, and hormones, have been explored as immunomodulators due to their efficacy in modulating immune pathways [15,16,17]. Antimicrobial peptides (AMPs) play a role in host defense and are promising therapeutic agents [18]. AMPs are small, making them easily degradable, and because of their antimicrobial properties, they are toxic to producer bacteria, hindering the production of recombinant AMPs [19]. Previous works have explored the activity of AMPs, such as defensin-5, lingual antimicrobial peptides [17], and multiple antimicrobial protein domains [20] in the formation of inclusion bodies (IBs). Cathelicidins are a group of AMPs with antibacterial and immunomodulatory functions that have been implicated in wound healing and innate immune mechanisms in fish. In salmonids, two cathelicidins have been described [21] with antibacterial activity against diverse Gram-positive and Gram-negative bacteria, such as *Aeromonas salmonicida*, *Vibrio anguillarum*, *Yersinia ruckeri*, *Photobacterium damselae*, and *Lactococcus garvieae* [22,23,24]. Another AMP that has shown these properties is hepcidin, which also has antibacterial and immunomodulatory effects on salmonids [25,26,27]. Other important host defense molecules are cytokines that regulate the immune response and can establish communication between different cell populations [28]. Interleukin one-beta (IL1β) and tumor necrosis factor-alpha (TNFα) are proinflammatory cytokines with overlapping roles as regulators of inflammation [29]. In fish, TNFα has been associated with regulating leukocyte homing, proliferation, and migration [30], and IL1β has been shown to play a relevant role in inducing inflammation, which is an important aspect of tissue regeneration, and in the activation of lymphocytes and phagocytic cells [29]. In salmonids, hepcidin has shown antimicrobial activity against pathogens such as *Piscirikettsia salmonis*, *A. salmonicida*, *Aeromonas hydrophila*, and *Pseudomonas aeruginosa* [26,27]. Cytokines as IBs have been previously evaluated in fish, where TNFα was tested as an immunostimulant in rainbow trout and zebrafish (*Danio rerio*) [15,16], and in pigs, where IL1, IL6, IL8, and TNFα were tested as stimulators of the intestinal mucosa [31]. To the best of our knowledge, although AMP-based IBs have been described [18], no work on teleost IBs of AMPs has been published. Hence, treatment with host-derived molecules to enhance immune function is an attractive alternative. However, AMPs and cytokines present considerable drawbacks, mainly related to their low stability and short half-life as soluble proteins [32,33]; however, encapsulation is necessary to avoid their degradation, thus increasing their cost.

BacterialIBs are protein aggregates [34] formed during recombinant production processes in microbial expression systems such as bacteria [16]. They are nanoparticles with sizes ranging between 50 and 500 nm [35] that exhibit greater stability than their soluble counterparts and have biological activity and functional proteins that are slowly released from them [36]. In recent years, the use of nanostructured proteins as IBs has emerged as an alternative to address the difficulties associated with soluble protein administration [15,37]. The production of IBs has been studied in shake flasks and also in bioreactors, although in this last case only with the aim of recovering soluble proteins [34]. Protein aggregation is improved by stress, such as high temperature, pH shifts, or high feeding rate [38,39,40], and, consequently, these parameters tend to result in higher yields of IBs. The unregulated nature of shaken flasks, and lack of controlled pH or oxygen, often results in variation in the expression but not in the quality of the IBs [34].

This study aimed to determine whether Atlantic salmon AMPs and rainbow trout cytokines can be produced as IBs that are able to activate the immune system. The scaling-up of IBs production was also characterized by comparing two different scale systems. The results of this work provide insight into host immune molecules as immunomodulators and the feasibility of scaling this technology.

## 2. Materials and Methods

### 2.1. Nanostructured Recombinant Protein Design

Five nanostructured recombinant proteins were produced as bacterial IBs: two antimicrobial peptides of Atlantic salmon (*Salmo salar*), cathelicidin (CAMP; Uniprot Q49TU5), and hepcidin (HAMP; Uniprot B5X878); two proinflammatory cytokines of rainbow trout (*Oncorhynchus mykiss*): IL1β (Unirprot Q9YGD3 [41]) and TNFα (Uniprot Q5BMN3 [41]); and near-infrared fluorescent proteins (iRFPs), which were used as nonimmune-relevant control proteins. Clones for CAMP, HAMP, and iRFP were designed using ORF and pET22b (Novagen, Sigma-Aldrich, San Luis, MO, USA) via a strategy in which the periplasmic location signal was removed and the C-terminal HisTag was included. Clones were codon optimized for expression in *Escherichia coli*, synthesized by GenScript (Hong Kong, China), and subcloned and inserted into pET22b. Recombinant plasmids were transformed into *E. coli* BL21(DE3) pLysS (Thermo Fisher Scientific, Waltham, MA, USA). The IL1β and TNFα coding sequences were cloned and inserted into pET28a (Novagen), which included a HisTag, and expressed in *E. coli* Rosetta-gami (DE3) (Novagen), as previously published [41].

### 2.2. Production of Nanostructured Recombinant Proteins

The methods for producing and purifying the IBs were described previously [16]. Briefly, *E. coli* was cultured in Luria Bertani (LB) medium supplemented with ampicillin at 50 μg/mL (Winkler, Santiago, Chile). The growth and production kinetics were carried out in 2 L shake flasks with 500 mL of culture medium at 300 rpm and 37 °C in a Labtech LSI-3016R0 incubator (Labtech, Namyangju, Korea). Each condition was tested in biological triplicates. The inoculum used was obtained from a 250 mL shake flask with 50 mL of culture medium, which was inoculated from a cryovial stored at −80 °C and left to grow overnight. For the cultures with induction, an early induction strategy was defined using 1 mM IPTG (Merck, Darmstadt, Germany), a biomass of 0.5 OD_600nm_, and a period of recombinant protein production that was extended for 3 h post-induction. IBs were isolated through enzymatic and mechanical disruption of the cells as described previously [31]. Briefly, bacterial cultures were incubated with lysozyme at 1 μg/mL (Winkler), phenylmethanesulfonyl fluoride (PMSF, Roche Diagnostic, Mannheim, Germany) at 0.4 mM was added, and the mixture was incubated at 37 °C for 2 h and 250 rpm. The samples were sonicated for 3 min (10 s on, 10 s off at 30% amplitude; Ultrasonic processor GE505) and stored at −80 °C overnight. Then, the suspension was frozen at −80 °C, thawed, Triton X-100 (0.2% (*v*/*v*); Winkler) was added, and the mixture was incubated at room temperature for 1 h with gentle agitation. The suspension was centrifuged, and the pellet was resuspended in phosphate-buffered saline (PBS; HyClone, Cytiva, Boston, MA, USA) using the original culture volume. Next, the samples were incubated with DNAse at 0.6 μg/mL (Roche Diagnostic) at 37 °C for 1 h at 250 rpm. Finally, the IBs were subjected to a sterility test without antibiotics, and 100 µL of suspension was cultured on LB agar plates at 37 °C for 3 days if bacterial growth was observed several freeze/thaw cycles were carried out until no viable bacteria were detected. Then, the IBs were purified and stored at −80 °C until use. The proteins were quantified via Western blotting using an anti-His-tag antibody (Invitrogen, Thermo Fisher Scientific), and the protein concentration was calculated from a standard curve generated using recombinant proteins and the ChemiDoc Imaging Systems MP (Bio-Rad Laboratories, Hercules, CA, USA).

IBs were characterized at nearly native state by field emission scanning electron microscopy (FESEM) operating at 2 kV and equipped with a secondary electron detector. The samples were resuspended in distilled water at a concentration of 100 mg/mL. Drops of 4 µL of IBs samples were directly deposited on silicon wafers (Ted Pella Inc., Redding, CA, USA) for 30 s and immediately observed without coating with the FESEM Merlin (Zeiss, Oberkochen, Germany). FESEM images were analyzed using the software ImageJ 1.54f (National Institute of Health, Bethesda, MD, USA) size measures of 300 nanoparticles per sample were taken.

### 2.3. In Vitro Analysis of the Modulation of the Immune Response

The RTS11 monocyte/macrophage line of *O. mykiss* (kindly donated by Dr. Niels Bols, University of Waterloo, Waterloo, ON, Canada) was cultured at 18 °C in Leibovitz’s L-15 medium (Gibco, Thermo Fisher Scientific) supplemented with 15% fetal bovine serum (FBS; Gibco) in 24-well plates at a density of 100,000 cells per well.

Cultured RTS11 cells were stimulated with different IBs to evaluate the expression of genes related to the immune response through RT–qPCR. For this purpose, we performed a time course and dose–response analysis. First, the cells were stimulated with CAMP^IB^, HAMP^IB^, or IL1β^IB^ at 5 µg/mL for 0 h (control), 4 h, 12 h, or 24 h. In a second experiment, the cells were incubated for 12 h with 10 µg/mL LPS (lipopolysaccharides from *Escherichia coli* O111:B4; Sigma-Aldrich) as a positive control, iRFP^IB^ (10 µg/mL) as an IBs control, CAMP^IB^, HAMP^IB^, or IL1β^IB^ at different concentrations (5, 10, and 20 µg/mL) or a control without stimulation. Due to the low concentration of TNFα^IB^ used during production, we ruled out the use of this protein. Each treatment was performed in triplicate. Total RNA was recovered using TRIzol^®^ (Invitrogen) following the manufacturer’s instructions. The RNA was quantified using a NanoDrop^TM^ device (Thermo Fisher Scientific, Waltham, MA, USA), and the integrity of the RNA was analyzed by visualizing the 28S and 18S rRNA bands resolved in 1.5% agarose gels stained with SBYR-safe DNA gel stain (Invitrogen). cDNA synthesis was performed using a RevertAid kit (Thermo Fisher Scientific) according to the manufacturer’s instructions. In addition, RT–qPCR was performed with SYBR Green (KAPA SYBR^®^ FAST, Merck) using an AriaMX real-time PCR thermocycler (Agilent, Santa Clara, CA, USA). The expression of several genes related to the immune innate profile was analyzed according to the nature of the proteins, i.e., an AMP or cytokine: interleukin one-beta (*il1β*), tumor necrosis factor-alpha (*tnfα*), interleukin eight (*il8*), interleukin ten (*il10*), interleukin 6 (*il6*), inducible nitric oxide synthase (*inos*), and cyclooxygenase 2 (*cox2*) (Table S1). Elongation factor 1 alpha (*ef1α*) and beta-actin (*β-actin*) were used as reference genes (Table S1), and quantification was performed according to the Livak method [42]. All samples were run in triplicate.

### 2.4. Scale-Up in a Bioreactor

To characterize the scale-up of IBs production, we compared the culture results between the shake flask and stirred tank bioreactor. The *E. coli* clones of IL1β were cultured in a New Brunswick Bioflo 115 bioreactor (Eppendorf, Hamburg, Germany) with a working volume of 1.5 L at 500 rpm, 1 vvm, and 37 °C. The non-induced cultures were cultured without pH control, and the pH change was monitored online using a Meter Toledo sensor (Meter Toledo, Columbus, OH, USA). The bioreactor cultures adhered to a similar methodology employed in the shake flask, except for pH regulation, which was set at a value of 7.0. Induction was executed at density of 0.5 OD_600nm_, and the duration of recombinant protein production was extended by an additional 3 h post-induction. A second production series was conducted in triplicate within the bioreactor, wherein induction was carried out at a cellular density of 1.0 OD_600nm_. The downstream process was carried out by moving 500 mL of cultivation broth to a shake flask in an incubator to undergo the same IBs purification procedure as that described previously. Finally, the treated culture broth was stored at −80 °C for storage and subsequent treatment and analysis of proteins in IBs. Purification and quantification were performed as described previously (Section 2.2).

### 2.5. Gene Expression Analysis

We compared the expression of immune-related genes induced in RTS11 by IL1β^IB^ produced in a shake flask or bioreactor by RT–qPCR. The cells were incubated for 12 h with 10 µg/mL LPS as a positive control, iRFP^IB^ (10 µg/mL) as an IBs control, or IL1β^IB^ produced in a shake flask or bioreactor at 10 µg/mL or a control without stimulation. Each treatment was analyzed in quintuplicate. As mentioned, total RNA, cDNA, and RT–qPCR were performed, and the details are described in Section 2.3.

### 2.6. Statistical Analysis

A non-parametric Mann–Whitney test was performed to analyze IBs size between treatments. For gene expression analysis, Kruskal–Wallis tests and Dunn post hoc tests were used to determine the fold change in expression. The gene expression data are expressed as the mean ± standard deviation (SD). For analysis of the growth parameters between the two scale productions, two key tests with the Holm–Šídák method were used. Significant differences are indicated when * *p* < 0.05 and ** *p* < 0.01. All the statistical analyses were performed, and graphs were generated using R software (version 4.1.2) and GraphPad Prism (version 10.1.0; GraphPad Software, Inc., San Diego, CA, USA). Regarding R software, we used the following packages: readxl (version 1.4.1), readr (version 2.1.3), ggplot2 (version 3.4.0), survminer (version 0.4.9), dplyr (version 1.0.10), tidyr (version 1.2.1), psych (2.2.9), lmtest (version 0.9-40), knitr (1.41), car (version 3.1-1), stats (version 4.1.2), multcompView (version 0.1-8), grid (version 4.1.2), gridExtra (version 2.3), cowplot (version 1.1.1), performanceAnalytics (version 2.0.4), onewaytests (version 2.6), lme4 (version 1.1-31), ggpubr (version 0.5.0), rstatix (version 0.7.1), pander (version 0.6.5), tidyverse (version 1.3.2), mdthemes (version 0.1.0) and openxlsx (version 4.2.5.1).

## 3. Results

### 3.1. Production of Inclusion Bodies

We successfully produced all the immune-related proteins from the salmonids in *E. coli* as IBs (Figure S1) in the following yields after purification: CAMP^IB^, 36.5 mg/L; HAMP^IB^, 28.5 mg/L; IL1β^IB^, 39 mg/L; and TNFα^IB^, 2 mg/L. Due to the low concentration of TNFα^IB^ used during production, we ruled out the use of this protein. The production efficiency of the iRFP^IB^ used in this study as a control has already been published [43].

### 3.2. In Vitro Analysis of the Modulation of the Immune Response by IBs

**Figure 1:**
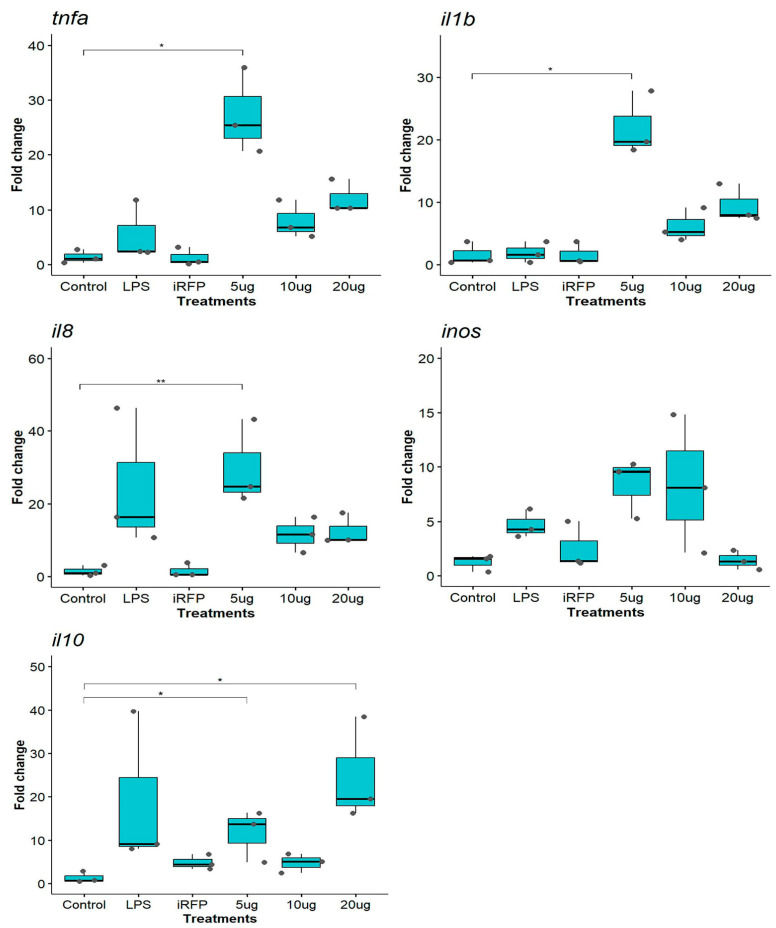
Gene expression analysis of the RTS11 cell line stimulated with CAMP^IB^. The cells were incubated for 12 h as follows: unstimulated control cells (control); LPS (10 µg/mL) as a positive control; iRFP^IB^ (10 µg/mL) as an immunogenically irrelevant IBs control; and CAMP^IB^ at three different concentrations (5, 10, and 20 µg/mL). The data are presented as the means ± SDs (n = 3), and the dots represent each of the data. Gene expression was determined by RT–qPCR, and the relative expression is represented as the fold change. Differences between the treatment means and controls were analyzed by the Kruskal–Wallis and Dunn tests. Significance levels: * *p* < 0.05; ** *p* < 0.01.

The short-term effects of CAMP^IB^, HAMP^IB^, and IL1β^IB^ on the modulation of immune markers were evaluated in the RTS11 cell line. First, the cells were stimulated with CAMP^IB^, HAMP^IB^, or IL1β^IB^ at 5 µg/mL for 0 h (control), 4 h, 12 h, or 24 h. The results showed that CAMP^IB^ and HAMP^IB^ significantly upregulated *tnfα* gene expression at 12 h (*p* < 0.05) (Figures S2 and S3). For genes such as *il1β*, the *inos* and *il10* data showed similar gene expression upregulation but no significant differences (Figures S2 and S3). For IL1β^IB^, we observed slight upregulation of *tnfα* at 4 and 24 h post-stimulation, and of *il1b*, *inos*, and *il10* at 4 h post-stimulation, although these differences were not significant compared with those of the control (Figure S3). Based on these results, we decided to explore the dose–response at 5, 10, or 20 µg/mL for 12 h. We used 10 µg/mL LPS as a positive control for immune response stimulation and 10 µg/mL iRFP^IB^ as a nonimmune-relevant control protein. The expression of innate immune response markers was analyzed via RTq–PCR (Figure 1). CAMP^IB^ upregulated the expression of all the genes analyzed. The results showed that, compared with the control, the smallest dose of CAMP^IB^ (5 µg/mL) significantly increased the expression of the proinflammatory cytokine *tnfα* (27.30 ± 7.82-fold change, *p* < 0.05). The 10 and 20 µg/mL doses did not follow a dose–response pattern; at the 10 µg/mL concentration, the expression decreased, and at 20 µg/mL, the expression of *tnfα* slightly increased. Interestingly, LPS and iRFP^IB^ treatments stimulated similar *tnfα* gene expression to that of the control. For *il1β*, we observed a similar pattern; the lowest dose of 5 µg/mL significantly upregulated *il1β* expression (21.97 ± 5.13-fold change, *p* < 0.05), and LPS and iRFP^IB^ treatments stimulated similar gene expression to that of the control (Figure 1). The expression of *il8* was significantly upregulated at 5 µg/mL (29.87 ± 11.66-fold change, *p* < 0.01), which was different from the findings for the other genes; moreover, LPS treatment significantly increased *il8* expression. Like the control, iRFP^IB^ stimulated *il8* gene expression. We did not observe significant differences in *inos* expression, although the lowest dose of CAMP^IB^ induced the highest expression (8.36 ± 2.70-fold change). We also assessed the expression of *il10*, an anti-inflammatory cytokine. The results showed that, compared with the control, the smallest and highest doses of CAMP^IB^ (5 and 20 µg/mL) significantly increased the expression of the genes (16.67 ± 2.89-fold change and 24.34 ± 10.85-fold change; respectively; *p* < 0.05), although the different doses evaluated did not follow a dose-dependent response.

**Figure 2:**
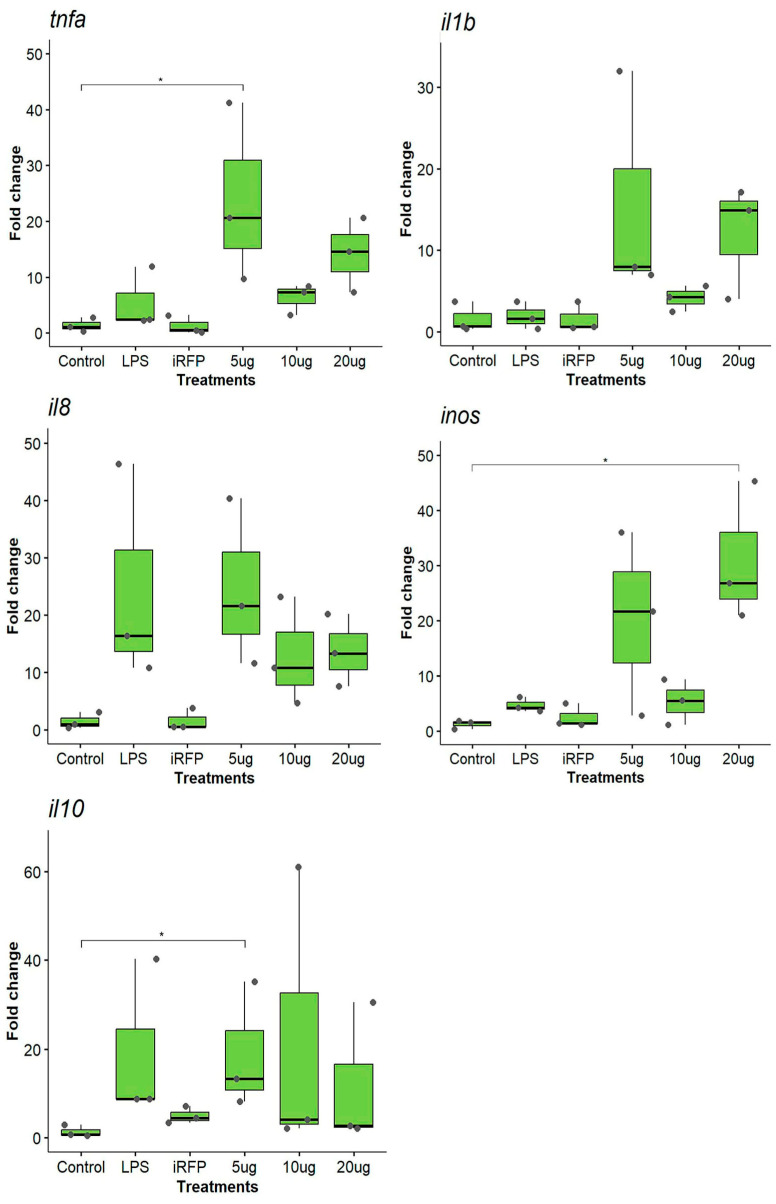
Gene expression analysis of the RTS11 cell line stimulated with HAMP^IB^. The cells were incubated for 12 h as follows: unstimulated control cells (control); LPS (10 µg/mL) as a positive control; iRFP^IB^ (10 µg/mL) as an immunogenically irrelevant IBs control; and HAMP^IB^ at three different concentrations (5, 10, and 20 µg/mL). The data are presented as the means ± SDs (n = 3), and the dots represent each of the data. Gene expression was determined by RT–qPCR, and the relative expression is represented as the fold change. Differences between the treatment means and controls were analyzed by the Kruskal–Wallis and Dunn tests. Significance levels: * *p* < 0.05.

For HAMP^IB^, we analyzed the same genes as for CAMP^IB^. Like what was observed for CAMP^IB^, the lowest dose of HAMP^IB^ produced the greatest upregulation of the expression of almost all the genes (Figure 2). For *tnfα*, we observed that only 5 µg/mL HAMP^IB^ significantly increased the expression of this proinflammatory cytokine (27.30 ± 7.82-fold change, *p* < 0.05). As for CAMP^IB^, the different doses analyzed did not follow a dose–response pattern, and iRFP^IB^ stimulated an expression pattern similar to that of the control (Figure 2). The data did not show significant upregulation of *il1β* expression after any treatment; however, we observed a substantial increase in the expression of *il1β* at 20 µg/mL (Figure 2). The results showed an upregulation of *il8* at the different doses; a greater increase was observed with 5 µg/mL (24.49 ± 14.58-fold change), although the difference was not significant. In the case of *inos*, we observed significant upregulation at 20 µg/mL (31.00 ± 12.67-fold change, *p* < 0.05) and a strong increase at 5 µg/mL, although these differences were not significant (Figure 2). At the immune regulatory level, *il10* expression increased significantly at 5 µg/mL (18.86 ± 11.66 fold change, *p* < 0.05); however, the different doses evaluated did not show a dose–response pattern. For CAMP^IB^ and HAMP^IB^, upregulation of the immune response of the genes evaluated was superior to that produced by iRFP^IB^, indicating that the stimulation is produced by the recombinant protein forming the IBs.

**Figure 3:**
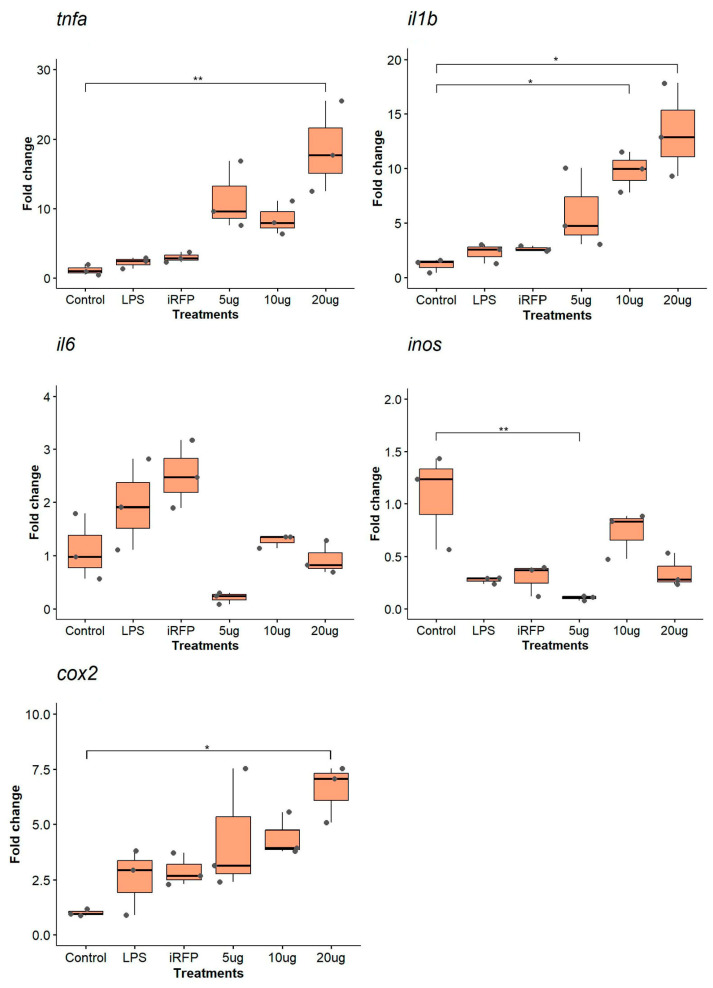
Gene expression analysis of the RTS11 cell line stimulated with IL1β^IB^. The cells were incubated for 12 h as follows: unstimulated control cells (control); LPS (10 µg/mL) as a positive control; iRFP^IB^ (10 µg/mL) as an immunogenically irrelevant IBs control; and IL1β^IB^ at three different concentrations (5, 10, and 20 µg/mL). The data are presented as the means ± SDs (n = 3), and the dots represent each of the data. Gene expression was determined by RT–qPCR, and the relative expression is represented as the fold change. Differences between the treatment means and controls were analyzed by the Kruskal–Wallis and Dunn tests. Significance levels: * *p* < 0.05; ** *p* < 0.01.

As for the proinflammatory cytokine IL1β^IB^, we analyzed the gene expression of a similar group of genes that responded to the characteristics of the protein to be evaluated (Figure 3). For *tnfα*, we observed that only 20 µg/mL IL1β^IB^ significantly increased the expression of this proinflammatory cytokine (18.56 ± 6.56-fold change, *p* < 0.01). Results showed a significant upregulation of *il1β* expression after treatment with IL1β^IB^ at 10 and 20 µg/mL (4.10 ± 1.32-fold change, and 4.87 ± 0.76-fold change, *p* < 0.05). The data did not show significant upregulation of *il6* expression after any treatment; in fact, *il6* expression was lower than in LPS and iRFP^IB^ treatments (Figure 3). For *inos*, we observed a significant downregulation at 5 µg/mL IL1β^IB^ (0.11 ± 0.02-fold change, *p* < 0.01). Finally, we analyzed *cox2* expression, where data showed a significant upregulation at 20 µg/mL (6.51 ± 1.31-fold change, *p* < 0.05).

### 3.3. Scale-Up in a Bioreactor

#### 3.3.1. Analysis of the Growth Parameters in Shake Flasks and Bioreactors

**Figure 4:**
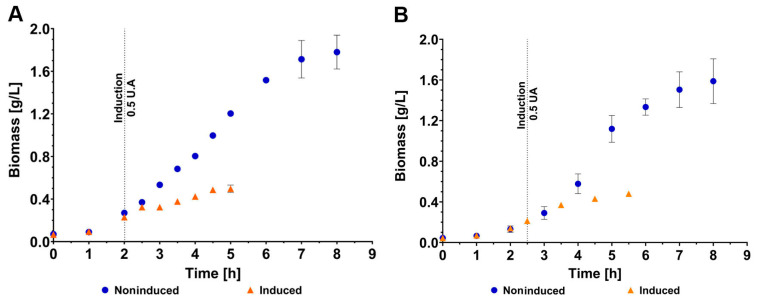
Growth profiles of *E. coli* Rosetta-gami (DE3) non-induced and induced by IPTG. (**A**) Shake flasks were shaken (500 mL) at 300 rpm and 37 °C. (**B**) Stirred tank bioreactors (1.5 L) at 500 rpm, 37 °C, 1 vvm, and pH controlled at 7.0, with induction at 0.5 U.A, 1.0 U.A, and not induced. The error bars represent the standard deviation, and the vertical lines represent the induction times.

To evaluate the scale-up in the bioreactor, we used the IL1β clone because previous studies have obtained this cytokine as an IBs and demonstrated its ability to induce an immune response in pigs [32]. Our first goal was to evaluate the kinetic profile of non-induced and induced *E. coli* Rosetta-gami (DE3) in cultures grown on two different production scales: 2 L shake flasks with 500 mL of medium (Figure 4A) and a 2 L bioreactor with 1.5 L of medium (Figure 4B). Previously, the inoculum size was set to a concentration of 50 mg/L (Figure S5), and induction with IPTG was performed once a concentration of 0.22 g/L (0.5 OD_600nm_) was reached.

The growth profiles of the non-induced cultures in shake flasks and in the bioreactor were similar, which ruled out an effect of the production scale on the kinetic profiles of the obtained *E. coli* Rosetta-gami (DE3) strains (Table 1). Similar biomass concentrations were detected in the shake flasks and bioreactors (1.78 ± 0.16 and 1.59 ± 0.22 g/L, respectively); however, in the bioreactor, there was a slight increase of 13% in the specific growth rate (0.63 ± 0.03 and 0.72 ± 0.02 h^−1^, respectively; *p* < 0.05). In the induced cultures, a drastic decrease in cell growth was observed, reaching a maximum biomass concentration of only 30% of that obtained without induction (0.50 ± 0.04 v/s 1.78 ± 0.16 g/L, *p* = 0.0001, and 0.48 ± 0.01 v/s 1.59 ± 0.22 g/L, *p* < 0.001; Table 1). No significant differences were observed between the maximum biomass concentration achieved with induction in shake flasks and bioreactors.

#### 3.3.2. IL1^IB^ Production in Shake Flasks and Bioreactors

To evaluate the production of the recombinant protein, the concentration of IL1β^IB^ as an IBs was analyzed after 3 h of induction (conditions optimized in a previous study [16]) for the two production scales used. In this sense, a 63% decrease in the production of IL1β^IB^ in the bioreactor was observed with respect to the strategy developed in shake flasks (39 ± 1.15 and 14.5 ± 4.08 mg/L, *p* < 0.001; Table 1). In terms of net production, the larger volume of culture carried out in the bioreactors allows a similar total mass to be generated in the shake flasks with respect to that in the bioreactor (19.5 ± 0.58 and 21.75 ± 6.12 mg/batch, respectively).

#### 3.3.3. Characterization of IL1β^IB^ by FESEM and Immunostimulation of RTS11 Cell Line

**Figure 5:**
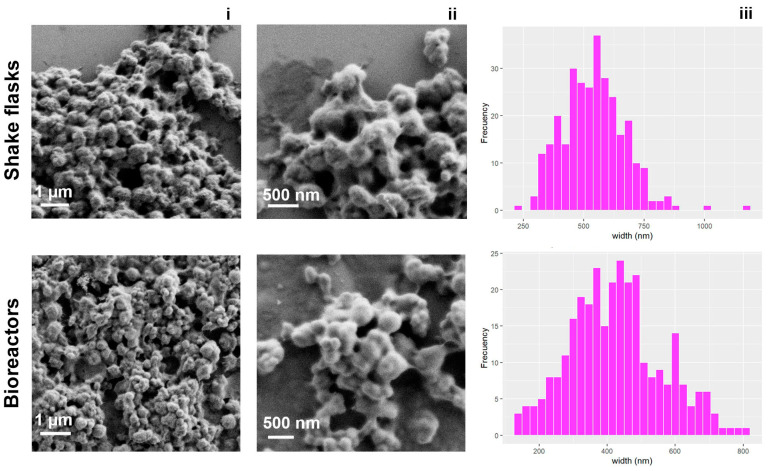
Characterization of the IL1β^IB^ protein. Field emission scanning electron microscopy (FESEM) images of IL1β^IB^ produced in shake flasks and bioreactors (**i**,**ii**). Size distribution histograms (n = 300; (**iii**)) of the IL1β^IB^ proteins.

To characterize the IBs obtained in the two production systems, we visualized them through field emission scanning electron microscopy (FESEM). IBs obtained in shake flasks and bioreactors maintained similar morphologies with round shapes and irregular surfaces. However, the data showed a significant difference in the sizes of the IBs produced with these methods; in the shake flasks, the IBs were 540 ± 129 nm in size, while in the bioreactor, they were 427 ± 134 nm in size (*p* < 0.0001; Figure 5).

**Figure 6:**
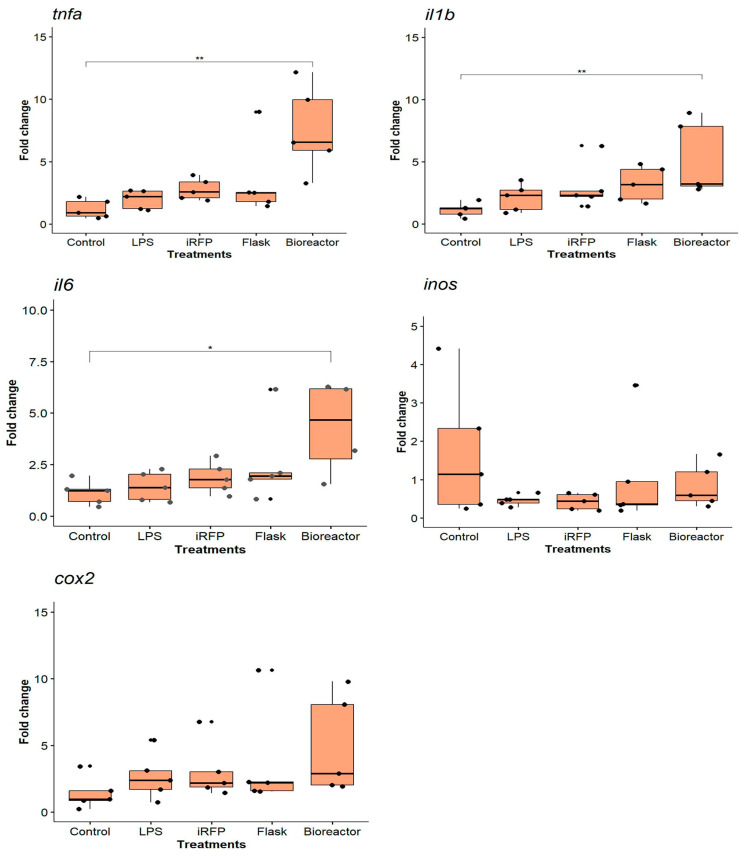
Gene expression analysis of the RTS11 cell line stimulated with IL1β^IB^. The cells were incubated for 12 h as follows: unstimulated control cells (control); LPS (10 µg/mL) as a positive control; iRFP^IB^ (10 µg/mL) as an immunogenically irrelevant IBs control; and Il1β^IB^ (10 µg/mL) produced in a shake flask and bioreactor. The data are presented as the means ± SDs (n = 5) and the dots represent each of the data. Gene expression was determined by RT–qPCR, and the relative expression is represented as the fold change. Differences between the mean values of the treatment groups and the control group were analyzed by the Kruskal–Wallis and Dunn tests. Significance levels: * *p* < 0.05; ** *p* < 0.01.

We investigated whether IL1β^IB^ produced in shake flasks or bioreactors could differentially affect the triggering of an immune response in the RTS11 cell line. We incubated IL1β^IB^ produced in shake flasks (F-IL1β^IB^) or in a bioreactor (B-IL1β^IB^) at 10 µg/mL for 12 h and analyzed the immune response by RT–qPCR. The results showed that, in general, B-IL1β^IB^ strongly upregulated immune genes compared with F-IL1β^IB^ (Figure 6). We observed that, compared with the control, B-IL1β^IB^ significantly upregulated the expression of *tnfα* (7.57 ± 1.92 and 1.98 ± 0.01-fold change, respectively; *p* < 0.01). Similarly, for *il1β*, B-IL1β^IB^ significantly upregulated its expression compared to that in the control group (4.62 ± 0.01 and 1.48 ± 0.44-fold change, respectively; *p* < 0.01). F-IL1β^IB^ upregulated *il1β* expression but not significantly. For *il6*, a difference in stimulation between IBs produced in the shake flask and in the bioreactor was also observed, where B-IL1β^IB^ was significantly upregulated compared to that in the control (7.05 ± 1.62 and 1.12 ± 0.01-fold change, respectively; *p* < 0.05). In the case of *inos* and *cox2*, we observed a tendency toward increased upregulation of these genes between B-IL1β^IB^ and F-IL1β^IB^; however, statistical analysis revealed no significant differences (Figure 6). For instance, *cox2* expression in B-IL1β^IB^ had a 4.92 ± 1.35-fold change, F-IL1β^IB^ had a 3.63 ± 0.97-fold change, *inos* expression in B-IL1β^IB^ had a 0.83 ± 0.02-fold change, and F- IL1β^IB^ had a 1.05 ± 0.02-fold change (Figure 6).

#### 3.3.4. Strategy for IL1β^IB^ Production in a Bioreactor with Late Induction

**Figure 7:**
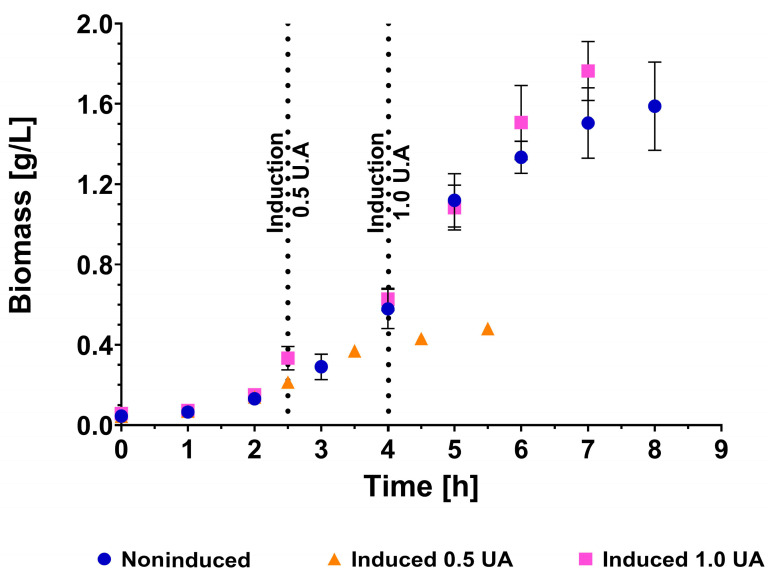
Comparison of the kinetic profile of IL1β^IB^ for the production of *E. coli* Rosetta-gami (DE3) in a bioreactor with 1.5 L of LB medium at 500 rpm, 37 °C, and pH 7.0. In blue fermentation without induction, orange fermentation was performed with early induction, and in pink fermentation, late induction. The error bars represent the standard deviation of the sample, while the dotted lines represent the time at which the induction was performed for each respective case.

Since the IBs produced under the controlled conditions of a bioreactor show promising results but low concentrations are reached, we proceeded to perform a second induction strategy considering induction at half of the exponential phase (0.44 g/L or 1.0 U/A) and keeping the other operational conditions constant (temperature, agitation, inductor concentration, induction time, etc.). The results showed that this production strategy allowed us to recover the growth profile without induction, during which the biomass concentration reached 1.51 ± 0.18 g/L and the specific growth rate was 0.73 ± 0.04 h^−1^ (Figure 7, Table 1). In addition, the production of recombinant protein in IBs increased to a concentration of 71.8 ± 5.2 mg/L, which implies an increase of four times the production achieved with early induction in a bioreactor and of 85% with respect to that achieved in shake flasks.

An interesting aspect is the specific productivity achieved. In this regard, slightly different specific productivities were observed in the bioreactor depending on the time of induction (10.1 ± 2.8 mg^IB^/g_cel_·h for early induction and 15.1 ± 1.1 mg^IB^g_cel_·h for late induction), while in shake flasks, values of 26.0 ± 0.77 mg^IB^/g_cel_·h were reached. This indicates that the increase in the production of IBs caused by late induction is a reflection of the greater number of producing cells present; thus, their characteristics should be maintained since the environmental conditions are equivalent to those of early induction.

## 4. Discussion

The rapid growth of aquaculture must be accompanied by measures to reduce the negative impact of infectious diseases. In this regard, new prophylactic methods need to be developed. Thus, IBs are emerging as potential alternatives with attractive characteristics, such as high stability, a slower release profile, and no need for encapsulation.

We observed that the two teleost IBs produced (CAMP^IB^ and HAMP^IB^) could modulate the immune response of the RTS11 cell line. In particular, CAMP^IB^ upregulated the expression of proinflammatory cytokines (*tnfα* and *il1β*), and chemokines (*il8*) at 5 µg/mL and of anti-inflammatory cytokines (*il10*) at 5 and 20 µg/mL. This could mean that *il10* modulates the inflammatory response because an exacerbated inflammatory state could cause detrimental effects on fish. This modulation was also observed because, at low *il10* gene expression, proinflammatory genes were still significantly upregulated at 5 µg/mL. Then, at a dose of 20 µg/mL, *il10* gene expression increased, and the expression of proinflammatory cytokines decreased. In mammals, cathelicidins play a role as mediators of immune activation and control of inflammation [44]. Cathelicidin promotes chemotaxis through IL8 in immune cells [44] and upregulates the gene expression of *tnfα* in the RAW264.1 cell line [45]. Additionally, it has been shown to induce the upregulation of *il8* and *il1β* gene expression in peripheral blood lymphocyte (PBL) cells, the upregulation of *il1β* gene expression in the RTgutGC cell line, and the induction of interferon-gamma (*ifnγ*) in head kidney leukocytes (HKLs) cells [23,24,46,47].

HAMP^IB^ significantly increased the expression of *tnfα*, *inos*, and *il10*, and we observed a clear tendency toward increased upregulation of *il1β* and *il8* gene expression at 5 µg/mL. Gene expression analysis did not show a dose-dependent response; in fact, for the gene expression of some genes, HAMP^IB^ exhibited maximum upregulation at the lowest dose. This could indicate a hypersusceptibility reaction and a possible difference in function from that observed in CAMP^IB^. According to the literature, hepcidin may act as an immunomodulatory peptide in the absence of a pathogen, modulating a potentially harmful proinflammatory response [48]. In vitro, hepcidin has been shown to have modulatory effects on the RTS11 cell line by upregulating the gene expression of *tnfα*, *il1β*, and *il10* [49]; moreover, in primary cell culture of the fin tissue of Caspian trout, hepcidin increased the upregulation of *tnfα* and *il6* gene expression [50]. On the other hand, in vivo, hepcidin upregulated the gene expression of proinflammatory cytokines, such as *tnfα* and *il1β*, and of an anti-inflammatory cytokine, such as *il10*, in the intestine, spleen, and head kidney of European sea bass [51]. Our results revealed that the increase in *il1β* induced by hepcidin, which has been described to increase the amount of circulating hepcidin [52], could potentiate the effects of IBs. However, for CAMP^IB^ the *il10* gene was upregulated only at the lowest dose, suggesting that other anti-inflammatory genes could regulate the pro-inflammatory process in HAMP^IB^.

Among the cytokines tested, IL1β^IB^ significantly upregulated the gene expression of *tnfα*, *il1β*, and *cox2* in the RTS11 cell line at 20 µg/mL, and the trend toward a dose-dependent response demonstrated a different response profile than that of the IBs of AMPs. In the case of *inos* and *il6*, a regulatory process could exist that inhibits their expression at the tested doses; however, at higher bioactivity of the IBs, as obtained in the bioreactor, we observed a significant increase in *il6* gene expression. The pro-inflammatory effects of IL1β observed in this study are in agreement with those previously described. IL1-type cytokines have a main function in controlling pro-inflammatory reactions [53]. IL1β is a primary cytokine released from cells during the immune response and can promote the production of cytokines such as *tnfα* [54], *il8* [55], *il18* [56], and more of itself [57]. For instance, in vitro, trout recombinant IL1β promoted inflammation and leukocyte migration by regulating chemokines such as CXC motif chemokine ligand 8 (CXCL-8) [55]. Furthermore, recombinant trout IL1β induces further changes in macrophages in vitro [57].

The possibility of modulating the immune system to enhance the response against pathogens seems very attractive, as it would reduce the negative impact of diseases and reduce the use of chemical compounds as treatments. The production of recombinant immune-related proteins, such as IBs, has been explored in animal models; for example, cytokines (IL1β, TNFα, IL6, IL8, and CCL4) in fish and pigs [15,16,31], and metalloproteinase-9 (MMP9) in cows [58,59]. Our results prompted us to explore antimicrobial peptides and cytokine IBs as possible immunomodulators in salmonids. CAMP^IB^, HAMP^IB^, and IL1β^IB^ produced pro-inflammatory and anti-inflammatory responses at 12 h. Gene expression analysis demonstrated that IBs have biological activity and are more efficient than LPS in stimulating the proinflammatory response in the RTS11 cell line. Our results suggest possible regulatory feedback effects between proinflammatory and anti-inflammatory cytokines. However, additional studies are necessary to determine the timing of each type of response and the best dose of inclusion bodies that produce a favorable proinflammatory response for fish defense and a good anti-inflammatory response with a larger sample size to corroborate our findings due to the variability of the present data.

On the other hand, the productivity yield of the IBs was similar to that previously described for similar proteins, obtaining yields between 2 mg/L and 39 mg/L of IBs in a shake flask. Similar studies reported yields between 2 and 20 mg/L for immune-related IBs, such as TNFα and CCL4 [16]. Even though we obtained IBs from rainbow trout TNFα^IB^ (2 mg/L), which is a low concentration compared to other IBs, we decided not to work with this protein. However, a previous study designed and produced TNFα^IB^ at a higher concentration (18 mg/L), which could be related to the expression plasmid and bacterial strain used (pQE-30 and *E. coli* M15 (pREP4), respectively), and the TNFα protein design, which in this case excluded the transmembrane domain [16,60].

With respect to the production of IBs, as described by several authors, the concentration and quality of these IBs are affected and can be controlled by various environmental and process factors [61,62], such as temperature, pH, agitation speed, dissolved oxygen concentration, and induction time among others [38,39,40,63]. This is reflected in the differences observed between our results in shake flasks and bioreactors. In our case, the temperature condition was not modified throughout the fermentation or between the different scales of the process; thus, contrary to cases in which the induction of recombinant protein production was performed by a change in temperature [38,64], this was not a factor to be considered. Even so, considering that it is desirable to obtain IBs with biological activity, we proceeded to perform the cultures at a temperature of 37 °C, which, according to various authors, is considered a thermal stress condition that favors the production of these IBs [65].

Our results reflect variations depending on the scale of the process (shake flasks and bioreactor). We observed a decrease in the concentration and specific productivity of the IBs obtained at a larger scale (bioreactor), which is not an expected result considering that the induction was performed at the same biomass concentration. The decrease in the concentration of IBs obtained may be because the controlled operating conditions of the bioreactor release the cells from certain stress conditions and because the cellular machinery is more amenable to correct folding of the heterologous protein during formation, increasing the production of the soluble fraction of the protein of interest. This differs in part from that reported by Restrepo-Pineda and collaborators [64], who obtained a higher concentration of IBs in bioreactors, although the specific productivity also decreased. These differences are partly associated with the production strategies used, while we used a standard complex medium (LB), they used a defined medium with glucose as the carbon source (17 g/L), which translates into potential cultures of low and high cell density. In the latter, oxygen transfer becomes a critical process parameter limiting the cell growth capacity and productivity of the process [63,66,67,68].

In this way, the transfer to a bioreactor allows a more efficient supply of oxygen and alleviates the limitations presented in shake flasks by generating more IB-producing biomass. In our case, the LB medium and early induction limited biomass generation; therefore, the transfer of available oxygen was not a critical process parameter under these conditions. On the other hand, in both cases, a decrease in the specific productivity of IBs was observed at the bioreactor scale, which can be attributed, as mentioned above, to the fact that the controlled environmental conditions allowed by the bioreactor favor the production of soluble protein.

On the other hand, the pH conditions between the shake flasks (without control) and the bioreactor (controlled at 7.0) had effects similar to those observed in the literature [39,40,61,69]. Without pH control in shake flasks, a greater concentration of IBs was generated, and a greater hydraulic diameter was observed (Figure 5); moreover, the controlled pH conditions in the reactors generated fewer IBs, which were smaller but had greater and longer immunogenic activity in the RTS11 cell line (Figure 6). This finding is in agreement with the results of Castellanos-Mendoza and collaborators, in which the IBs that formed at free pH reached sizes greater than 500 nm, while at controlled pH, the sizes were less than 500 nm. Moreover, these authors demonstrated that at controlled pH, IBs presented greater resistance to denaturing agents, similar to what was reported by Calcines-Cruz and collaborators [39]. This could explain why our IBs obtained in the bioreactor presented longer immunogenic activity than the other IBs in the RTS11 cell line. Considering that to increase the production of IL1β in IBs different strategies can be addressed such as the addition of more substrate, or different modes of operation such as batch-fed, the confirmation of these findings allows us to lay a good foundation for the production for therapeutic purposes of our IBs.

The application of late induction (in the middle of the exponential phase) in the bioreactor allowed a 4-fold increase in the concentration of the protein obtained (71.8 mg/L) and a slight increase in the specific productivity. These results are promising, given that, as reported by Upadhyay and collaborators [70], this parameter strongly correlates with the quality and characteristics of IBs; therefore, we can infer that it was possible to increase the concentration of IBs without affecting their immunogenic activity. Moreover, these findings are consistent with several reports in which the concentration and characteristics of IBs are controlled by the rate of consumption of carbon sources, maintaining a constant specific production [71,72].

Several aspects warrant further investigation, including the modification of the chemical induction method by IPTG, such as exploring temperature induction. Additionally, there is a need for the design and optimization of a defined medium specifically tailored for the production of IBs. Investigating strategies like fed-batch production in high-density bacterial culture could offer valuable insights. Optimization of bacterial lysis through mechanical methods is another aspect that could be explored. Defining an extraction and purification strategy compatible with a pilot-scale process is essential to reduce production costs and streamline the scaling-up process.

## 5. Conclusions

In summary, this study successfully demonstrated the production of functional antimicrobial peptides and a cytokine in the form of IBs from salmonids and rainbow trout using *E. coli* as the expression host. The proteins, CAMP^IB^, HAMP^IB^, and IL1β^IB^, exhibited promising yields after purification, and in vitro analysis revealed that they effectively modulated the immune response in the RTS11 cell line. The present study examined the short-term effects on the expression of various immune markers and demonstrated that CAMP^IB^, HAMP^IB^, and IL1β^IB^ could upregulate proinflammatory and anti-inflammatory cytokines, suggesting regulatory feedback between them and indicating their potential as immunomodulators.

Scaling up the production to bioreactors was explored, focusing on IL1β^IB^. The growth parameters in shake flasks and bioreactors were analyzed, and the production of IL1β^IB^ in bioreactors exhibited a 63% decrease compared to shake flasks, suggesting the impact of controlled environmental conditions on IBs production. Late induction in bioreactors was implemented, resulting in a 4-fold increase in IL1β^IB^ concentration compared to early induction. This strategy also maintained specific productivity, indicating a potential avenue for enhancing IBs production without compromising quality. The study provides valuable insights into the production, characterization, and modulation of immune responses by host immune molecule IBs. Future research directions may include optimizing production conditions, exploring alternative induction strategies, and assessing the applicability of these IBs as immunomodulators in aquaculture for disease prevention. The findings contribute to the growing field of recombinant protein production for therapeutic and biotechnological applications, particularly in the context of sustainable aquaculture.

## Figures and Tables

**Table 1:** Growth profile of the IL1β^IB^ culture in shake flasks and bioreactors.

Parameters	Shake Flask		Bioreactor		
	Non-Induced	Induced at 0.5 U.A	Non-Induced	Induced at 0.5 U.A	Induced at 1.0 U.A
Growth velocity (µ) (h^−1^)	0.63 ± 0.03	-	0.72 ± 0.02	-	0.73 ± 0.04
Culture time	8	5	8	5.5	7
Time to raise induction biomass (h)	-	2	-	2.5	4
Biomass raised (g/L)	1.78 ± 0.16	0.50 ± 0.04	1.59 ± 0.22	0.48 ± 0.01	1.51 ± 0.18
Biomass raised at harvest time (g/L)	1.20 ± 0.02	0.50 ± 0.04	1.12 ± 0.13	0.48 ± 0.01	1.51 ± 0.18
IBs Yields (mg/L)	-	39 ± 1.15	-	14.5 ± 4.08	71.8 ± 5.2
Total IB (mg)	-	19.5 ± 0.58	-	21.75 ± 6.12	107.7 ± 7.8
IB specific productivity (mg/g·h)	-	26.0 ± 0.77	-	10.1 ± 2.8	15.1 ± 1.1

## Data Availability

The raw data supporting the conclusions of this article will be made available by the authors, without undue reservation, to any qualified researcher.

## Supplementary Materials

The following supporting information can be downloaded at: https://www.mdpi.com/article/10.3390/ani14060844/s1, Table S1. Primers used for RTq-PCR. Figure S1: IBs detection by Western blotting using an anti-His-tag antibody. Figure S2. Gene expression analysis of the RTS11 cell line stimulated with CAMP^IB^. Figure S3. Gene expression analysis of the RTS11 cell line stimulated with HAMP^IB^. Figure S4. Gene expression analysis of the RTS11 cell line stimulated with IL1β^IB^. Figure S5: Effect of inoculum size on the growth of *E. coli* Rosetta-gami (DE3) in flasks shaken at 300 rpm and 37 °C with free pH; Figure S6. IBs detection by Western blotting using an anti-His-tag antibody. CAMP^IB^, HAMP^IB^, IL1β^IB^, and TNFα^IB^ were detected through ChemiDoc Imaging Systems—uncropped blots.
