# Supplementary material for: Immunomodulation Evidence of Nanostructured Recombinant Proteins in Salmonid Cells
Source: Animals (Basel). 2024 Mar 9;14(6):844. doi: 10.3390/ani14060844 (PMC10967322; doi:10.3390/ani14060844)
Supplement: Supplementary file 1 [file animals-14-00844-s001.zip › animals-2869585-supplementary.pdf]

animals

MDPI

Supplementary Materials

Table S1. Primers used for RTq-PCR.

Target Gene	Sequence 5'to 3`	Tm (Cº) Annealing	Efficiency (%)	Accession Number	Reference
ef1α	Fw: CAAAGCCCTGAAGTCTGGAG<br>Rv: GACTTGGTCACCTTGCCAGT	60<br>60	95	NM_001141909.1	
B-actin	Fw: CCCATCTACGAGGGTTACGC<br>Rv: ACTCCTGCTTGCTGATCCAC	62<br>62	100	XM_014194536.2	Valenzuela et al., 2023*
tnfα	Fw: GCAGCCATCCATTTAGAGGGTGAA<br>Rv: CTAAACGAAGCCTGGCTGTAAACG	60<br>60	100	DQ787157.1	Valenzuela et al., 2023*
il10	Fw: ATGAGGCTAATGACGAGCTGGAGA<br>Rv: GGTGTAGAATGCCTTCGTCCAACA	60<br>60	91	XM_045705802.1	
il8	Fw: GCCCTCCTGACCATTACTGA<br>Rv: AAATCTCCTGACCGCTGTTG	60<br>60	101	NM_001140710.3	Valenzuela et al., 2023*
il1β	Fw: GTCACATTGCCAACCTCATCATCG<br>Rv: GTTGAGCAGGTCCTTGTCCTTGA	60<br>60	100	NM_001123582.1	
cox2	Fw: CAGTGCTCCCAGATGCCAAG<br>Rv: GCGAAGAAGGCGAACATGAG	60<br>60	95	XM_014139742.2	
il-6	Fw: AGTTTCAGAAGCCCGTGGAAGA<br>Rv: GGGGAGTAGGGTTGATTGAGGGTA	60<br>60	100	XM_014143031.2	
inos	Fw: CCAGCATAAGTGGTTCCAAGACCT<br>Rv: CCAATCTCAGTGCCCATGTACCAG	60<br>60	96	XM_035787944.2	

(*) Valenzuela, C.A.; Azúa, M.; Ojeda, N.; Álvarez, C.A.; Schmitt, P.; Mercado, L. Evidence of the Autophagic Process during the Fish Immune Response of Skeletal Muscle Cells against Piscirickettsia salmonis. Animals 2023, 13, 880. https:// doi.org/10.3390/ani13050880

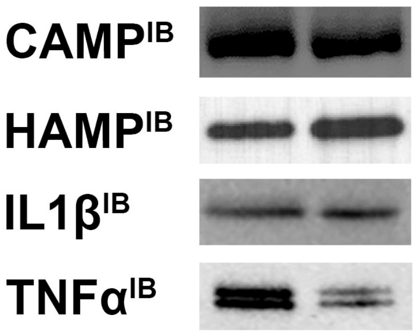

Figure S1. IBs detection by western blotting using an anti-His-tag antibody. CAMP^{IB}, HAMP^{IB}, IL1β^{IB} and TNFα^{IB} were detected through ChemiDoc Imaging Systems.

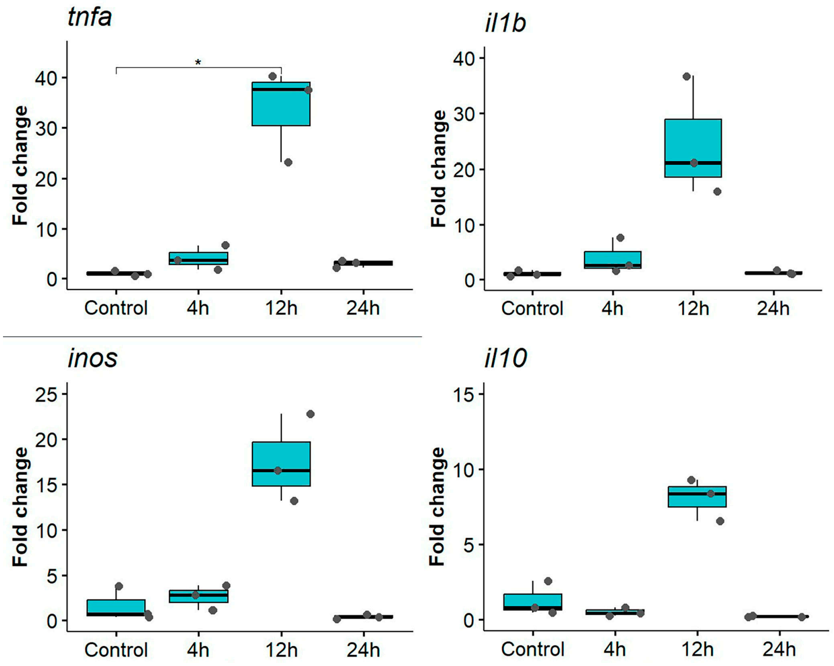

Figure S2. Gene expression analysis of the RTS11 cell line stimulated with CAMP[IB] at 5 μg/ml. The cells were incubated for 4 h, 12 h and 24 h. Control treatment is time 0 h. The data are presented as the means ± SDs (n= 3), and the dots represent each of the data. Gene expression was determined by RT–qPCR, and the relative expression is represented as the fold change. Differences between the treatment means and controls were analyzed by the Kruskal–Wallis and Dunn tests. Significance level: * $p < 0.05$.

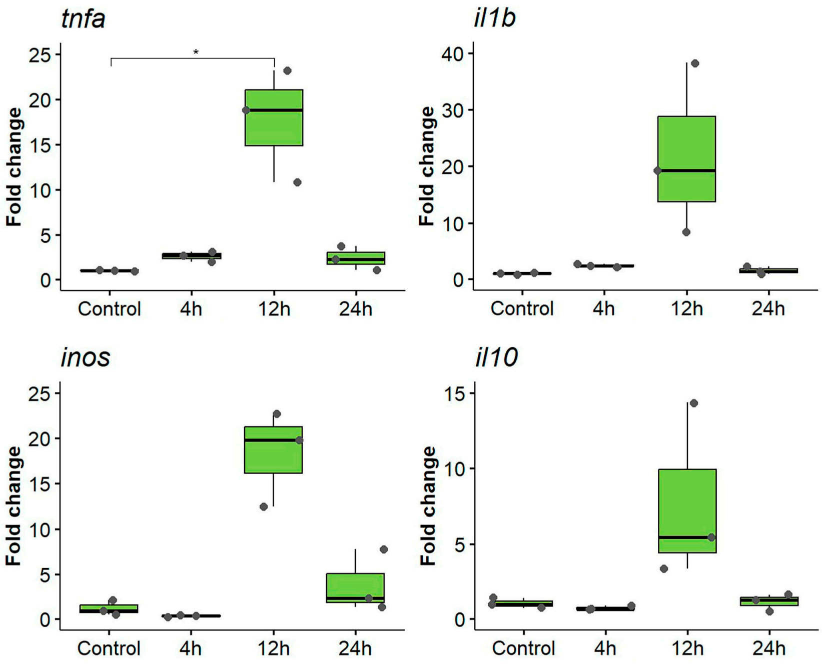

Figure S3. Gene expression analysis of the RTS11 cell line stimulated with HAMP[IB] at 5 μg/ml. The cells were incubated for 4 h, 12 h and 24 h. Control treatment is time 0 h. The data are presented as the means ± SDs (n= 3), and the dots represent each of the data. Gene expression was determined by RT–qPCR, and the relative expression is represented as the fold change. Differences between the treatment means and controls were analyzed by the Kruskal–Wallis and Dunn tests. Significance level: * $p < 0.05$.

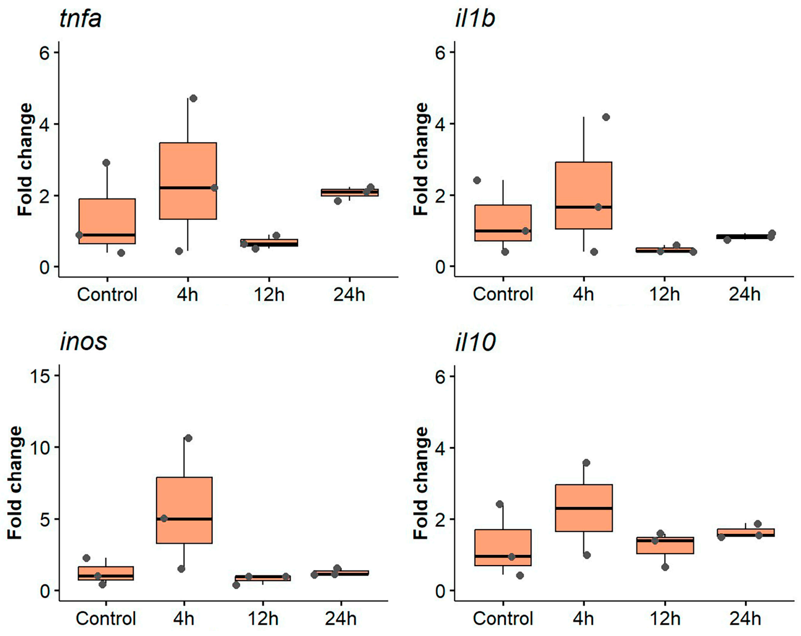

Figure S4. Gene expression analysis of the RTS11 cell line stimulated with IL1β^{IB} at 5 µg/ml. The cells were incubated for 4 h, 12 h and 24 h. Control treatment is time 0. The data are presented as the means ± SDs (n= 3), and the dots represent each of the data. Gene expression was determined by RT–qPCR, and the relative expression is represented as the fold change. Differences between the treatment means and controls were analyzed by the Kruskal–Wallis and Dunn tests.

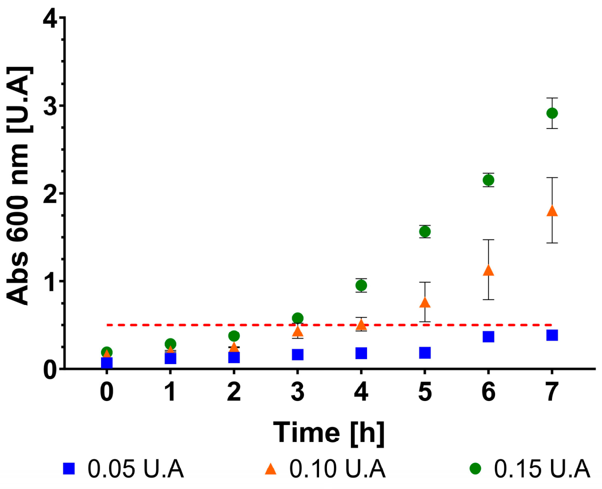

Figure S5. Effect of inoculum size on the growth of *E. coli* Rosetta-gami (DE3) in flasks shaken at 300 rpm and 37°C at the appropriate pH. The blue square represents the 0.05 U.A., the orange triangle represents the 0.10 U.A., the green circle represents the 0.15, and the red line represents the concentration at which induction was subsequently performed (0.5 U.A.). The error bars represent the standard deviation of the samples (n= 3).

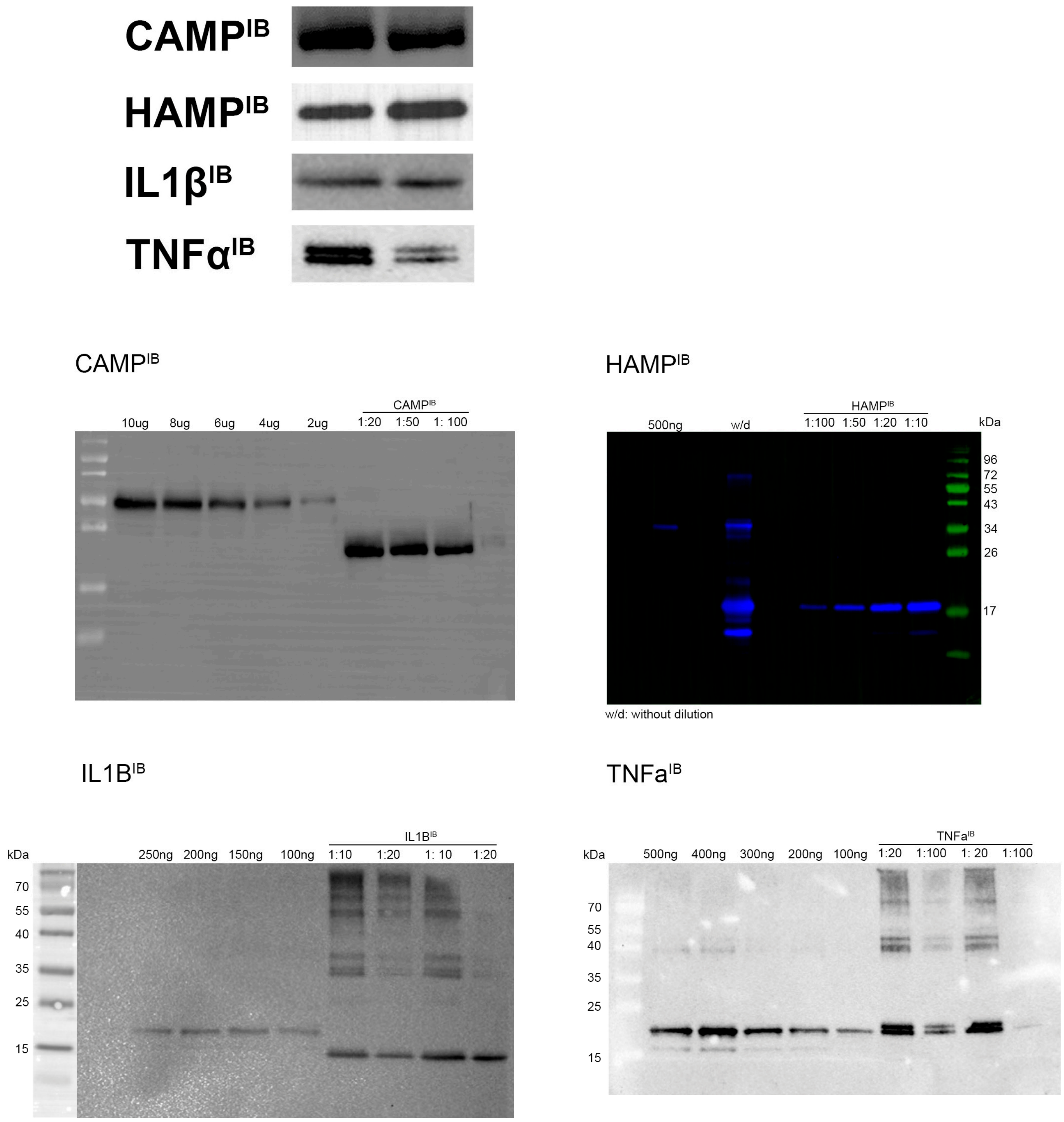

Figure S6. IBs detection by western blotting using an anti-His-tag antibody. CAMP^{IB}, HAMP^{IB}, IL1β^{IB} and TNFα^{IB} were detected through ChemiDoc Imaging Systems – uncropped blots.
